# Putative Nonribosomal Peptide Synthetase and Cytochrome P450 Genes Responsible for Tentoxin Biosynthesis in *Alternaria alternata* ZJ33

**DOI:** 10.3390/toxins8080234

**Published:** 2016-08-02

**Authors:** You-Hai Li, Wen-Jin Han, Xi-Wu Gui, Tao Wei, Shuang-Yan Tang, Jian-Ming Jin

**Affiliations:** 1 Beijing Key Laboratory of Plant Resources Research and Development, Beijing Technology and Business University, Beijing 100048, China; lyhlf11@sina.com (Y.-H.L.); 15632685950@163.com (W.-J.H.); weitao.1220@163.com (T.W.); 2 Key Laboratory of Ethnic Medicine Resource Chemistry, State Ethnic Affairs Commission & Ministry of Education, Yunnan Minzu University, Kunming 650500, China; 3 CAS Key Laboratory of Microbial Physiological and Metabolic Engineering, Institute of Microbiology, Chinese Academy of Sciences, Beijing 100101, China; guixiwu123@hotmail.com (X.-W.G.); tangsy@im.ac.cn (S.-Y.T.)

**Keywords:** *Alternaria alternata*, tentoxin, nonribosome peptide synthetase, cytochrome P450

## Abstract

Tentoxin, a cyclic tetrapeptide produced by several *Alternaria* species, inhibits the F_1_-ATPase activity of chloroplasts, resulting in chlorosis in sensitive plants. In this study, we report two clustered genes, encoding a putative non-ribosome peptide synthetase (NRPS) TES and a cytochrome P450 protein TES1, that are required for tentoxin biosynthesis in *Alternaria alternata* strain ZJ33, which was isolated from blighted leaves of *Eupatorium adenophorum*. Using a pair of primers designed according to the consensus sequences of the adenylation domain of NRPSs, two fragments containing putative adenylation domains were amplified from *A. alternata* ZJ33, and subsequent PCR analyses demonstrated that these fragments belonged to the same NRPS coding sequence. With no introns, *TES* consists of a single 15,486 base pair open reading frame encoding a predicted 5161 amino acid protein. Meanwhile, the *TES1* gene is predicted to contain five introns and encode a 506 amino acid protein. The TES protein is predicted to be comprised of four peptide synthase modules with two additional *N*-methylation domains, and the number and arrangement of the modules in TES were consistent with the number and arrangement of the amino acid residues of tentoxin, respectively. Notably, both *TES* and *TES1* null mutants generated via homologous recombination failed to produce tentoxin. This study provides the first evidence concerning the biosynthesis of tentoxin in *A. alternata*.

## 1. Introduction

**Figure 1:**
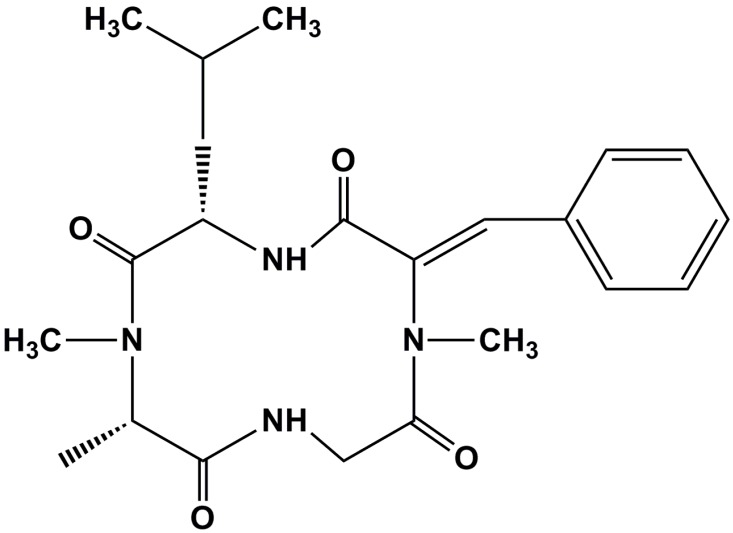
Structure of tentoxin.

*Alternaria* is a genus of common filamentous fungi that includes many saprophytic and plant pathogenic species. *Alternaria* produce a variety of toxic secondary metabolites, such as tentoxin, altenuene, alternariol, alternariol methyl ether, altertoxins, and tenuazonic acid, which play an important role in fungal pathogenicity and are harmful to both humans and animals [1]. *A. alternata*, previously known as *A. tenuis* Nees *auct*, is an important plant pathogen that causes a variety of diseases in higher plants, including leaf spots, rots, and blights. *A. alternata*, as well as *A. citri*, *A. longipes*, *A. mali*, *A. porri*, and *A. tenuis* [2,3,4,5,6], are known to produce tentoxin (Figure 1), a natural cyclic tetrapeptide [cyclo-(L-MeAla-L-Leu-Me∆^Z^Phe-Gly)] that damages the F_1_-ATPase of chloroplasts in many sensitive plants, thereby inducing chlorosis, making it a potential selective herbicide [7]. The mechanism of tentoxin inhibition at low concentration is due to its capability to block the activity of the F_1_-ATPase in chloroplasts of sensitive plants [8,9].

Cyclic peptides comprise a large family of structurally diverse natural products that includes many important pharmaceutical antibiotics such as actinomycin, bacitracin, cephalosporin, gramicidin, penicillin, and vancomycin, as well as the antitumor agent bleomycin and the immunosuppressive agent cyclosporin A [10,11,12]. In addition to the classical 20 amino acid residues found in proteins, cyclic peptides contain a large number of unusual non-proteinogenic amino acid residues. Nonribosomal peptide synthetases (NRPSs) are a class of large multi-enzyme complexes that catalyze the synthesis of natural cyclic peptides in microorganisms. NRPSs consist of several modules. Each complete module contains approximately 1100 amino acid residues and consists of three domains, including a condensation domain (C), an adenylation domain (A), and a thiolation domain (T); however, the first module typically lacks a condensation domain. Meanwhile, some modules may contain extra domains such as an epimerization domain (E), a heterocyclization domain (Cy), and an *N*-methylation domain (M). Each module is usually specific to a particular amino acid substrate, and the extra domains modify the incorporated amino acid. Moreover, such modifications are often essential for bioactivity [10,13].

Several NRPS genes have been reported in *Alternaria* species. The cyclic peptide synthetase gene *AMT* was shown to be involved in AM-toxin synthesis in *A. alternata* [14], while the HC-toxin synthetase gene *HTS1* is involved in the biosynthesis of the HC-toxin in *A. jesenskae* [15]. Two other NRPS genes, *AbrePsy1* and *AbNPS2*, were detected in *A. brassicae*. While the function of *AbrePsy1* has yet to be characterized [16], *AbNPS2* was found to play an important role in the development and virulence of *A. brassicae* [17]. Recently, De Bruyne Lieselotte et al. reported that the NRPS gene *CmNps3* is responsible for tentoxin biosynthesis in *Cochliobolus miyabeanus*, and predicted that *AaNps3* could be involved in tentoxin biosynthesis in *Alternaria* species [18]. However, there has been no evidence reported on tentoxin synthetase genes in *Alternaria*.

In this study, we isolated *Alternaria* strains from blighted leaves of *Eupatorium adenophorum*, and successfully cloned the NRPS genes from the tentoxin-producing strain *A. alternata* ZJ33. A putative NRPS gene (*TES*) and a cytochrome P450 gene (*TES1*) required for tentoxin biosynthesis were identified via targeted gene mutagenesis, and by chemotype analyses of the resulting mutants. This study provides the first report on two genes involved in tentoxin biosynthesis in *A. alternata* ZJ33. Characterization of these tentoxin biosynthesis genes will further our understanding of the detailed mechanism of tentoxin biosynthesis in *A. alternata*. Moreover, characterization of the tentoxin biosynthesis genes in *A. alternata* will likely contribute to the functional characterization of similar genes in other fungi.

## 2. Results

### 2.1. Identification of Isolate ZJ33

Fifty-two endophytic fungal strains were isolated from leaves of *E. adenophorum*. Of these, ITS sequence of isolate ZJ33 showed 100% identity to that of *A. alternata* strain HZ1111 and ZG-2-3-2. Isolate ZJ33 was initially light grey in color and changed to dark green, and then black after 3 days of incubation on potato dextrose agar (PDA) at 25 °C. The surface of isolate ZJ33 was black when PDA plate was completely covered by branched and septate mycelia. Conidiophores were light brown and septate with terminal conidia. Conidia were oval and bean shaped with 3–5 transverse septa and 0–3 longitudinal septa. Conidial dimensions varied from 9.2 to 3.5 μm in width and 21.8 to 32.5 μm in length. Based on these characters, isolate ZJ33 was identified as *Alternaria alternata* [19]. The identity of isolate ZJ33 was confirmed by China General Microbiological Culture Collection Center. The subsequent HPLC analysis demonstrated that this organism produced tentoxin in modified Richard’s solution at 25 °C for four weeks.

### 2.2. PCR Amplification of NRPS Gene Fragments

A pair of degenerate primers specific to conserved sequence motifs of NRPS genes (cps1 and cps2) was then used to PCR amplify NRPS gene fragments from the genomic DNA of *A. alternata* ZJ33, and PCR products of expected size were cloned into the pGEM^®^-T Easy vector. The resulting twelve plasmids containing putative NRPS sequences were subjected to nucleotide sequencing and BLASTX analysis using the NCBI database. Of the amplified sequences, the open reading frames of two NRPS gene fragments (Figures S1 and S2) from *A. alternata* ZJ33 exhibited the highest amino acid identity to the first (66% identity) and third adenylation domains (85% identity) of tentoxin synthase CmNPS3 of *C. miyabeanus* and NPS3 of *C. heterostrophus* [18,20], indicating that these two fragments are part of the same NRPS gene. Indeed, primers (NRPS-for and NRPS-rev) designed using the DNA sequences of the two NRPS gene fragments amplified a 7.5 kb product from the genomic DNA of strain ZJ33.

### 2.3. Characterization of TES and TES1

*TES* (Figure S3), which was predicted to contain no introns, consists of 15,486 base pairs (bp) and encodes a 5161 amino acid protein, with a calculated molecular mass of 576.6 kilodaltons (kDa). The *TES* open reading frame exhibited strong similarity to the tentoxin synthase CmNPS3 from *C. miyabeanus* and to the NPS3 coding sequence in *C. heterostrophus* (79% and 78% sequence identity, respectively) [18,20], as well as to a number of the other known NRPSs. Furthermore, detailed analysis of the amino acid sequence of TES revealed that the predicted protein exhibits typical NRPS modular organization. Specifically, TES was predicted to consist of four modules with additional *N*-methyltransferase domains in both the second and fourth modules. The domain arrangement of TES is as follows: A-T-C-A-M-T-C-A-T-C-A-M-T-C. The number and arrangement of the modules in TES were consistent with the number and arrangement of the amino acids of tentoxin, respectively.

**Figure 2:**
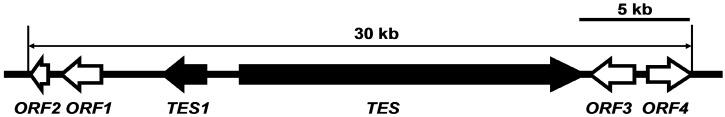
Gene organization of the 30 kb DNA region near two clustered genes required for tentoxin biosynthesis in *A. alternata* ZJ33. The arrow and arrowhead indicate a predicted gene and its transcriptional direction; black arrows represent the genes required for tentoxin biosynthesis. *ORF1–ORF4* encode four predicted proteins, respectively.

The *TES1* gene (Figure S4), which was closely linked to *TES* in a 5′ end-to-5′ end arrangement (Figure 2), was predicted to encode 1,837 bp and contain five introns. There is an 1.4-kb region of non-structural gene between *TES* and *TES1* (Figure S5). The predicted protein encoded by the *TES1* gene consists of 506 amino acid residues and has a molecular mass of 58.1 kDa, and exhibited 83% and 81% amino acid sequence identity to the hypothetical protein SETTUDRAFT_25251 of *Setosphaeria turcica* Et28A and to the hypothetical protein COCMIDRAFT_27438 of *Bipolaris oryzae* ATCC 44560 [21], respectively. Furthermore, TES1 contains a highly conserved amino acid sequence (WGYDNHVCPG) and was therefore predicted to be a member of the cytochrome P450 family.

### 2.4. Targeted Disruption of TES and TES1 in A. alternata ZJ33

**Figure 3:**
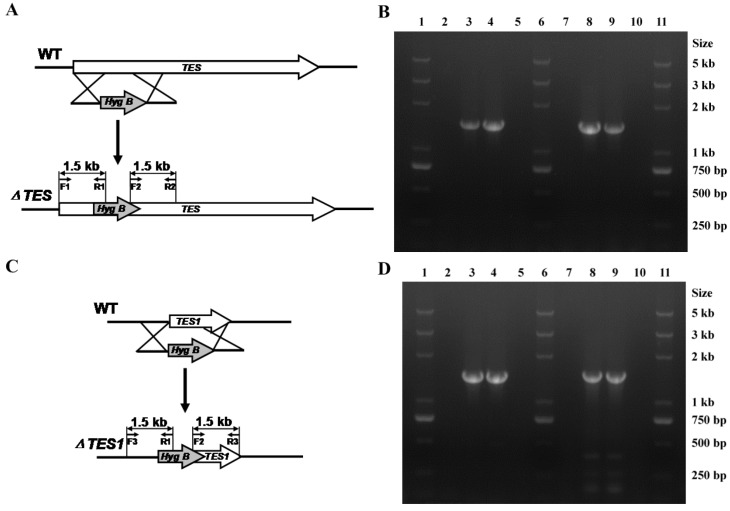
Disruption of the *TES* or *TES1* genes via homologous recombination. (**A**,**C**) Schematic diagram illustrating the targeted gene disruption strategy. WT, wild-type strain ZJ33; *ΔTES TES* null mutant; and *ΔTES1*, *TES1* null mutant. (**B**) Verification of *TES* disruption by PCR analysis. Lanes 2–5: PCR amplification was performed using primer pairs TES-5for (F1) + Hyg-5rev (R1); lanes 7–10: PCR amplification was performed using primer pairs Hyg-3for (F2) + TES-3rev (R2); lanes 1, 6, and 11: DNA ladder markers; lanes 2 and 7: wild-type strain ZJ33; lanes 3–4 and 8–9: *TES* null mutants; lanes 5 and 10: ectopic transformants. (**D**) Verification of *TES1* disruption by PCR. Lanes 2–5: PCR amplification was performed using primer pairs TES1-5for (F3) + Hyg-5rev (R1); lanes 7–10: PCR amplification was performed using primer pairs Hyg-3for (F2) + TES1-3rev (R3); lanes 1, 6, and 11: DNA ladder markers; lanes 2 and 7: wild-type strain ZJ33; lanes 3–4 and 8–9: *TES1* null mutants; lanes 5 and 10: ectopic transformants. A 1.5 kilobase (kb) band was expected to be amplified from the genomic DNA of both the *TES* and *TES1* null mutants by PCR. The sizes of standards are indicated on the right.

To disrupt the *TES* and *TES1* genes, we transformed double-joint PCR (DJ-PCR) products into *A. alternata* ZJ33, respectively. For targeted disruption of *TES*, a 430 bp fragment of *TES* was replaced with the fungal selectable marker *HygB*, which provides resistance to hygromycin B, via double homologous recombination (Figure 3A). To confirm disruption of *TES*, the genomic DNA of each resulting transformant was analyzed using two pairs of primers to distinguish *TES* null mutants from ectopic transformants (Figure 3B). Specifically, primer pairs designed to bind to a region of the *HygB* cassette and to regions of the *TES* gene fragment flanking the insertion site (TES-5for (F1) + Hyg-5rev (R1) and Hyg-3for (F2) + TES-3rev (R2)) successfully amplified the predicted 1.5 kb products, respectively, from the genomic DNA of *TES* null mutants but not from that of the wild-type strain ZJ33 or the ectopic transformants, thereby confirming the insertion of the *HygB* cassette (Figure 3B). Of ten randomly-chosen transformants, six were identified as *TES* null mutants while four were identified as ectopic transformants (Figure S6A,B). Identical methods were used for disruption of *TES1* and for identification of *TES1* null mutant (Figure 3C,D), and among ten randomly-chosen transformants, eight and two were identified as *TES1* null mutants and as ectopic transformants (Figure S6C,D), respectively.

**Figure 4:**
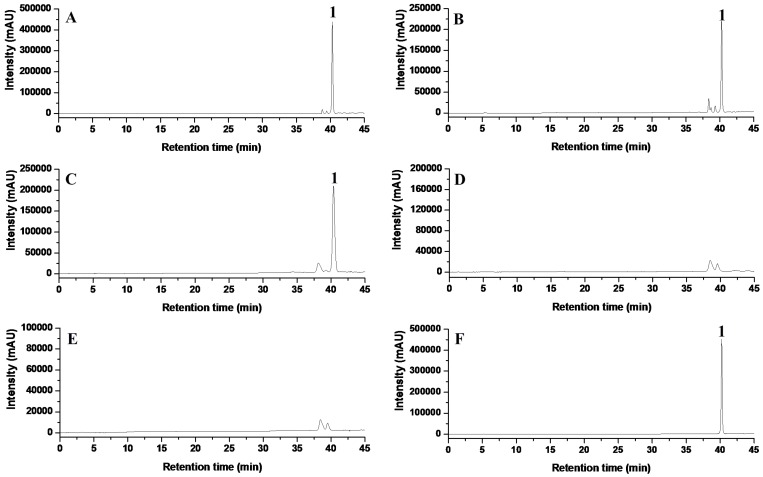
HPLC analysis of tentoxin from *A. alternata* ZJ33 and transformants: (**A**) *A. alternata* ZJ33; (**B**) *TES* ectopic transformant; (**C**) *TES1* ectopic transformant; (**D**) *TES* null mutant; (**E**) *TES1* null mutant; and (**F**) Standard tentoxin. Retention time of tentoxin (Peak 1): 40.3 min.

HPLC was subsequently utilized to detect tentoxin production in the mycelia of each strain. After culturing, a tentoxin peak was detected in the media harvested from the wild-type strain ZJ33, the *TES* ectopic transformant and the *TES1* ectopic transformant (Figure 4A–C), but not in those from the *TES* null mutants or the *TES1* null mutants (Figure 4D,E). Furthermore, the retention time of tentoxin was approximately 40.3 min.

## 3. Discussion

**Figure 5:**
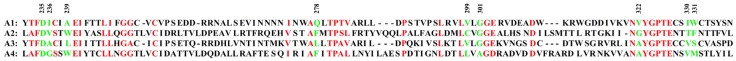
Putative binding-pocket constituents in the four adenylation domains of TES.

**Figure 6:**
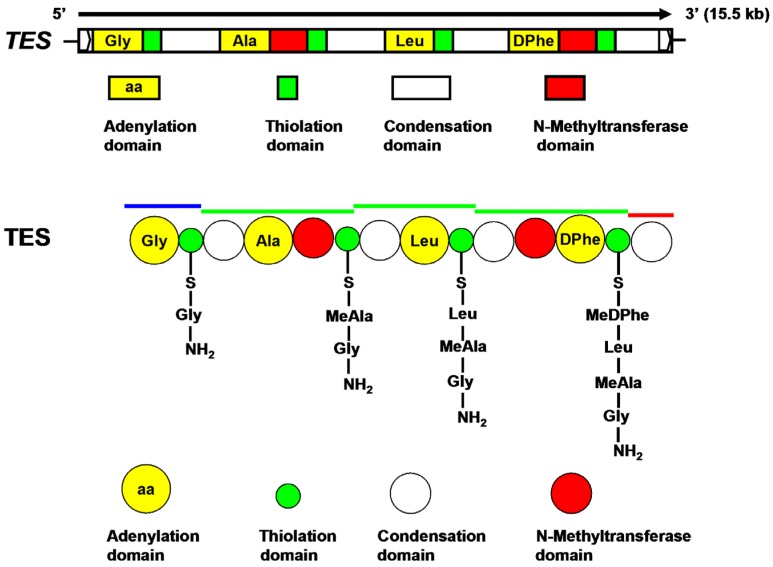
Schematic diagram of the modules in TES.

*A. alternata* is the most common *Alternaria* species isolated from *Eupatorium adenophorum* [22,23,24]. In this study, we therefore isolated *Alternaria* strains from *E. adenophorum* and demonstrated that *A. alternata* ZJ33 produced tentoxin, which was identified with HPLC and spectrometric determination (Figure 4A and Figure S7). Subsequently, we cloned and identified two clustered genes, *TES* and *TES1*, that were required for tentoxin biosynthesis in *A. alternata* ZJ33. Tentoxin is a cyclic tetrapeptide that consists of four amino acid residues: glycine (Gly), alanine (Ala), leucine (Leu), and dehydrophenylalanine (DPhe). In addition, both the Ala and DPhe residues are *N*-methylated. A previous study examining tentoxin biosynthesis and characterizing a tentoxin synthetase isolated from a strain of *A. alternata* suggested that tentoxin was biosynthesized by NRPS in *Alternaria* species [25,26]. Furthermore, the tentoxin synthetase of *A. alternata* was reported to be a polyfunctional protein without subunits. Recently, the NRPS CmNPS3 was identified to be tentoxin synthase in *C. miyabeanus* by gene knockout [18]. Using primers designed to recognize a conserved motif of NRPS genes, we cloned NRPS gene fragments from two *A. alternata* strains and found that only two NRPS fragments were amplified from the tentoxin-producing strain ZJ33. Signature sequences (the binding-pocket constituents) within adenylation domains are important for substrate recognition [27], and detailed analysis of the complete amino acid sequence of TES revealed the binding-pocket constituents of four putative adenylation domains (Figure 5 and Table 1). Thus, similar to tentoxin synthase CmNPS3 of *C. miyabeanus* and other NRPSs [18], TES assembles tentoxin from four substrate amino acids. According to the chemical structure of tentoxin and the binding-pocket constituents of the four putative adenylation domains of TES, we predicted that these putative adenylation domains might be responsible for the activation of Gly, Ala, Leu, and DPhe, respectively. In addition, two putative *N*-methyltransferase domains in the second and fourth modules of TES could be responsible for *N*-methylation of Ala and DPhe residues, respectively. Finally, we predicted that the condensation domain located in the termination module of TES catalyzes formation of the intramolecular macrocyclization and then the release of tentoxin product (Figure 6).

*TES1* null mutant strains failed to produce tentoxin, indicating that the cytochrome P450 protein TES1 was also required for tentoxin biosynthesis in *A. alternata*. As mentioned above, tentoxin contains the unusual aromatic amino acid residue DPhe. Meanwhile, the other three amino acid residues that comprise tentoxin (Gly, Ala, and Leu) are non-essential amino acid in fungi. Thus, *TES1* is predicted to be involved in DPhe biosynthesis. Cytochrome P450s catalyze oxidation of various substrates. The role of cytochrome P450s in the biosynthesis of natural products is versatile and function of many cytochromes P450 has not been revealed [28]. The substrate of TES1 has not been identified. In fungi, bacteria, and plant species, the shikimate pathway, or common aromatic pathway, is essential for the biosynthesis of the three aromatic amino acids and the aromatic secondary metabolites [29]. However, the mechanism of DPhe biosynthesis is currently unclear. While we presume that another gene(s) is involved in DPhe biosynthesis, the genomic regions flanking *TES* and *TES1* in *A. alternata* ZJ33 contain no obvious candidates that may contribute to this process, suggesting that such sequences may be located elsewhere in the genome.

We have proved that the two clustered genes, *TES* and *TES1*, are required for tentoxin biosynthesis in *A. alternata* ZJ33. Lieselotte De Bruyne et al. reported the function of *CmNps3* in *C. miyabeanus* and predicted the function of *AaNPS3* in *A. alternata* [18]. We have found that the two clustered genes in *A. alternata* strain SRC1lrK2f, OAG16164 and OAG16165 (GenBank), encode an NRPS and a cytochrome P450 monooxygenase-like protein which are similar to TES and TES1, respectively. In *C. miyabeanus*, there is also a cytochrome P450 gene near to *CmNps3*. Thus, the NRPS and the cytochrome P450 are conserved in *A. alternata* and *C. miyabeanus*.

In summary, we have identified two genes, *TES* and *TES1*, involved in tentoxin biosynthesis in *A. alternata*. These findings could prove useful for future studies investigating the functions of similar NRPS proteins in fungi. Future studies include the identification of the remaining biosynthetic machinery required for DPhe biosynthesis.

## 4. Experimental Section

### 4.1. Strains, Media, and Culture Conditions

*A. alternata* ZJ33 was isolated from blighted leaves of *E. adenophorum* collected in Lufeng County of Yunnan Province, China, in August 2012. *A. alternata* ZJ33 is available at China General Microbiological Culture Collection Center (CGMCC3.17853). *A. alternata* ZJ33 was stored in 20% glycerol at −80 °C or maintained on potato carrot agar (20 g potato, 20 g carrot and 20 g agar for 1 L). For genomic DNA isolation, the strains were grown in 50 mL YPG medium (20 g glucose, 10 g peptone and 5 g yeast extract for 1 L) at 28 °C for 72 h in a rotary shaker (Suzhou Peiying Experimental Equipment Co., Ltd., Suzhou, China) (180 rpm), and mycelia were harvested and lyophilized.

### 4.2. Nucleic Acid Manipulations

Fungal genomic DNA was extracted with a CTAB method as previously described [30]. Standard procedures were used for agarose gel electrophoresis, restriction endonuclease digestion and ligation. The PCR primers used in this study were synthesized by Life Technologies Co., Beijing, China. The PCR primers were dissolved in sterilized water to 100 μM and stored at −20 °C.

### 4.3. Identification of Fungi

rDNA from the ITS region of the isolated fungi was amplified via PCR with primers ITS4 and ITS5 [31,32]. PCR products were sequenced (Majorbio Bio-Pharm Technology Co., Ltd., Beijing, China). The sequence of ITS region has been deposited in NCBI Genbank under the accession number KX437754. The resulting sequences obtained were blasted against NCBI Genbank data.

### 4.4. Cloning of NRPS Genes from A. alternata ZJ33

A pair of degenerate primers cps1 and cps2, was designed based on the conserved core sequences in NRPS A domains (Table 2) [14]. Genomic DNA from *A. alternata* ZJ33 was used as templates. The thermocycler programme for PCR was set as: 94 °C for 2 min, 30 cycles of 94 °C for 1 min, 50 °C for 1 min and 72 °C for 1 min, and 72 °C for 5 min. The sizes of the PCR products were analyzed by 1% agarose gel electrophoresis and the expected DNA fragment was cloned into pGEM^®^-T Easy vector according to the manufacturer’s instructions (Promega (Beijing) Biotech Co., LTD., Beijing, China). Transformants were selected on LB agar plates supplemented with ampicillin (50 µg/mL). Plasmid DNAs were purified from 5 mL *E. coli* culture in LB medium (10 g tryptone, 5 g yeast extract and 10 g NaCl) supplemented with ampicillin (50 µg/mL), using a plasmid purification kit (Omega Bio-Tek Inc., Norcross, GA, USA). Inserts in the plasmids were verified by *Eco*R I digestion and PCR, then were sequenced.

### 4.5. Accession Numbers

Genomic DNA of *A. alternata* ZJ33 was sequenced by de novo genome sequencing. *TES* and *TES1* were cloned by PCR and sequenced again (Majorbio Bio-Pharm Technology Co., Ltd., Beijing, China). The sequences of *TES* and *TES1* have been deposited in NCBI Genbank under accession numbers KT947104 and KT947105, respectively.

### 4.6. Double-Joint PCR (DJ-PCR) for Constructing Mutant Alleles

To determine whether the cloned genes are functional for tentoxin biosynthesis in *A. alternata*, targeted gene disruption was performed by the DJ-PCR method. The 5′ and 3′ regions of each target gene were amplified from the genomic DNA of *A. alternata* ZJ33 with primer pairs specific to these regions (Table 2). A 1.5-kilobase (kb) *HygB* resistance gene fragment was amplified from the plasmid pCSN44 with primer pair *HygB*-for and *HygB*-rev [33]. The reverse primer for the 5′ region and the forward primer for the 3′ region carried 20-bp sequence tails (in italics) that overlap with both of the 5′ and 3′ ends of the *HygB* cassette, respectively. Three amplicons (5′ flanking region, *HygB*, and 3′ flanking region) were mixed and used as template for the second round of PCR, in which ten cycles were carried out without the addition of primers, followed by an additional PCR reaction (35 cycles) with the new nested primer pairs. Following phenol extraction and ethanol precipitation, the final PCR products (approximately 3.8 kb and 3.9 kb, respectively) were directly used for fungal transformation.

### 4.7. Targeted Mutagenesis

*TES* and *TES1* null mutants were generated by disrupting the targeted gene using homologous recombination, respectively (Figure 3A, C). Protoplasts were prepared from *A. alternata* ZJ33 as described previously with modifications [14,34]. *A. alternata* ZJ33 was cultured in YPG liquid medium at 28 °C for 48 h in a rotary shaker (180 rpm), and mycelia were harvested. Mycelia were suspended in 0.7 M NaCl containing 10 mg of driselase per mL (Sigma-Aldrich, St. Louis, MO, USA) and 10 mg of lysing enzymes per mL (Sigma-Aldrich, St. Louis, MO, USA), and placed in a rotary shaker (50 rpm) for 3 h at 30 °C in the dark to generate protoplasts. Transformation was performed as previously described [30]. Transformants were selected on PCA plates containing hygromycin B (50 µg/mL). *TES* and *TES1* null mutants were confirmed by PCR, respectively. Primers used to confirm the null mutants are showed in Table 2.

### 4.8. High-Performance Liquid Chromatography (HPLC) Analysis of Tentoxin

The wild-type strain ZJ33 and the transformants were grown in 50 mL of liquid seed medium (30 g sucrose, 5 g peptone, 2 g NaNO_3_, 1 g K_2_HPO_4_, 0.5 g KCl, 1 g MgSO_4_·7H_2_O and 0.02 g FeSO_4_·7H_2_O for 1 L) at 28 °C for three days in a rotary shaker (180 rpm). Then 10 mL of the cultured seed medium was transferred into 500 mL flasks with 200 mL of modified Richard’s solution (50 g glucose, 10 g KNO_3_, 5 g KH_2_PO_4_, 5 g MgSO_4_·7H_2_O, 0.02 g FeCl_3_, 0.005 g ZnSO_4_, 20 mL V8 vegetable juice for 1 L) and grown at 28 °C for four weeks.

The cultured medium was filtered and extracted with 200 mL of ethyl acetate. The ethyl acetate layer was concentrated to dryness and redissolved in methanol. Standard tentoxin was purchased from Sigma-Aldrich, Co., St. Louis, MO, USA. A Shimadzu LC-20AT HPLC system and a Symmetry C_18_ column (4.6 × 250 mm, Waters Corporation, Milford, MA, USA) were used to analyze tentoxin. The mobile phase was gradient aqueous methanol (increased from 20% to 100% in 45 min); flow rate was 0.4 mL/min; column temperature was 35 °C; and tentoxin was detected at 285 nm.

## Figures and Tables

**Table 1:** Signature sequences of amino acid in four putative adenylation domains of TES.

Domain	Position									Substrate
	235	236	239	278	299	301	322	330	331	
A1	D	I	A	Q	V	G	V	I	W	Gly
A2	D	V	W	F	C	G	G	T	F	Ala
A3	D	A	L	L	V	G	A	V	S	Leu
A4	D	G	W	F	L	A	A	V	M	DPhe

**Table 2:** Primers used in this study.

Primer ^a^	Sequence ^b^ (5′→3′)
ITS4	TCCTCCGCTTATTGATATGC
ITS5	GGAAGTAAAAGTCGTAACAAGG
cps1	AATCTAGATAYGGNCCNACNGA
cps2	CCTCTAGANAGRTCNCCNGTYTTR
NRPS-for	GAGGCAAGGCAACCGCAACGATGA
NRPS-rev	CCCTTCATGTCGGGACTTGCGACA
*HygB-*for	CCGGGCTGCAGGAATTCGAT
*HygB-*rev	GGATCCCGGTCGGCATCTAC
TES-5for	CGGGATCGCTACTGTTTGACGTCA
TES-5rev	*ATCGAATTCCTGCAGCCCGG*TTGCCTTGCCTCAGCCAGA
TES-3for	*GTAGATGCCGACCGGGATCC*GCTAAAGTGGACCGGCAGA
TES-3rev	CATCGATCCCGATTGGCGTTCACA
TES-5nest	GAAGATATGGGAGAGAAACCGCGG
TES-3nest	AATGGTGGCATCGTTCTGGCCAGT
P450-5for	ACCTGACGAAACTCACTGCCTCCT
P450-5rev	*ATCGAATTCCTGCAGCCCGG*CTCAGGCTCCATCTTTGGAG
P450-3for	*GTAGATGCCGACCGGGATCC*AGGGCCTGGAGGAAACCTACAC
P450-3rev	GCTCGACCATTGGATACCTAGCCT
P450-5nest	ACAATGGCCTAAGTCTGCCGCTCA
P450-3nest	CCCTCAACATTCCCTGGCATCTTG
Hyg-5rev	CGCACAAGTTATCGTGCACCAAGC
Hyg-3for	GGCGTATATGCTCCGCATTGGTCT

^a^ The name of target gene for disruption precedes the abbreviation of the primer names. -5for, the forward primer for amplification of the 5′-flanking region of a target gene; -5rev, the reverse primer for the 5′ region; -3for, the forward primer for the 3′ region; -3rev, the reverse primer for the 3′ region; -5nest, primer nested in -5′for; -3nest, primer nested in -3′rev; ^b^ The italic sequences are matched with the primer sequences of either *HygB-*for or *HygB*-rev.

## Supplementary Materials

The following are available online at www.mdpi.com/2072-6651/8/8/234/s1, Figure S1: >NRPS gene fragment 1 from *A. alternata* ZJ33; Figure S2: >NRPS gene fragment 2 from *A. alternata* ZJ33; Figure S3: >Seq1 (organism = *Alternaria alternata*) tentoxin synthase (TES) gene of *A. alternata* ZJ33, complete cds; Figure S4: >Seq2 (organism = *Alternaria alternata*) cytochrome P450 (TES1) gene of *A. alternata* ZJ33, complete cds; Figure S5: DNA sequences between *TES* and *TES1* (red); Figure S6: Verification of *TES* or *TES1* disruption by PCR analysis. lanes 1: DNA ladder markers; lanes 2–11: transformants; lane 12: wild-type strain ZJ33; (A) verification of *TES* disruption by PCR amplification using primer pairs F1 (TES-5for)+R1 (Hyg-5rev); lane 2, lanes 4–5, lane 7 and lanes 10–11: *TES* null mutants; lane 3, lane 6 and lanes 8–9: ectopic transformants; (B) verification of *TES* disruption by PCR amplification using primer pairs F2 (Hyg-3for)+ R2 (TES-3rev); lane 2, lanes 4–5, lane 7 and lanes 10–11: *TES* null mutants; lane 3, lane 6 and lanes 8–9: ectopic transformants; (C) verification of *TES1* disruption by PCR amplification using primer pairs F3 (TES1-5for) + R1 (Hyg-5rev); lane 2, lanes 4–9 and lane 11: *TES1* null mutants; lanes 3 and 10: ectopic transformants; (D) verification of *TES1* disruption by PCR amplification using primer pairs F2 (Hyg-3for) + R3 (TES1-3rev); lane 2, lanes 4–9 and lane 11: *TES1* null mutants; lanes 3 and 10: ectopic transformants; Figure S7. Absorbance spectrum of standard tentoxin and tentoxin from *A. alternata* ZJ33, (A) Standard tentoxin (Peak 1); (B) Tentoxin (Peak 1) from *A. alternata* ZJ33.
