# Supplementary material for: Putative Nonribosomal Peptide Synthetase and Cytochrome P450 Genes Responsible for Tentoxin Biosynthesis in Alternaria alternata ZJ33
Source: Toxins (Basel). 2016 Aug 2;8(8):234. doi: 10.3390/toxins8080234 (PMC4999850; doi:10.3390/toxins8080234)
Supplement: Supplementary file 1 [file toxins-08-00234-s001.pdf]

Supplementary Materials: Putative Nonribosomal Peptide Synthetase and Cytochrome P450 Genes Responsible for Tentoxin Biosynthesis in *Alternaria alternata* ZJ33

You-Hai Li, Wen-Jin Han, Xi-Wu Gui, Tao Wei, Shuang-Yan Tang and Jian-Ming Jin

GTGTAGCATTTGGTGCACTTCGTATTCCAATACCGACCGAGAATTCCGATCGGGAACGATAGGCACGTCAATGGCTTCTTT

TAGTTGGGTTACCGACCCGGAAGACCACAACAAGCTAGTGCCATTTGGCACAATTGGCGAACTTCTGATAGAGGGACCAAT

TCTGGCACGCGGGTACCTCAACGACATTTCGAAAACTGAGGCTGTTTTCGTTGATGGCCCTCTCTGGCTGAGGCAAGGCAA

CCGCAACGATGAATCCACTCGACGACAGGGAAGACTATA

Figure S1. >NRPS gene fragment 1 from *A. alternata* ZJ33.

GATCAACGCCTACGGGCCCACTGAATGTTGTGTCTCCTGTGTCGCAAGTCCCGACATGAAGGGACTCGATCCGGAACCCAT

TGGGAAGCCGATtGCGTCaATTGGCTGGGTGACTAATCCTAACGATCACAATAGACTAGCACCGCTAGGCGCTGTTGGAGA

GCTGTTGGTTGAGGGACCAAACCTAGCCCGCGGCTACCTGGACGATGCGAAGAAGACAGAGACAGCTTTTGTCCATGATCC

TCTCTGGCTGCTTCGGGGCTGTGAAGGCTACAGTGGGCGGCGAGGTAGACTGTACAAGACAGGCGACCTGGTCTACCACA

Figure S2. >NRPS gene fragment 2 from *A. alternata* ZJ33.

ATGCCGGGATCGCTACTGTTTGACGTCAACAAACTCGATGTCGAGAAGATATGGGAGAGAAACCGCGGACCTTTGCGAGC

GGTCAATCGCTGCGTTCACAGCCTATACGAAGAGCAAGCTATAGCTCGACCGAACGCATGCGCCATTCACGCATGGGACG
GCAACATGACCTACGAGCAATTGAATCAGCATTCAACACGATTGGCAAGCTATCTTGTCACTCAAGGCATTGGAACCGAG
GTGATGGTACCACTGTGCTTTGAGAAGTCGATATGGGCGATCGTAGCTATGTTAGCTGTTCTCAAGGCTGGTGCCGCGTT
CGTGCCACTAGACCCAATGCATCCAAGAGCCCGCCACGAAGAGATATTCAAGCAGACCAATGCGAAGTTGGTCCTTACTT
CCGTACAGCACGCCGCTCTCTGGCCAAATTCGGGTCTGCAGTTTCTGGCAATCGACAAAACCTTTGTTGATCAATTGCCA
TGGGAAACCAAGATCCGGTCCAAAGTCAAGCCAATAGATGCTGTCTATGTTATGTTCACATCTGGTAGTACTGGTGTTCC
GAAAGGCGTTGTCTTGGAGCATAGAGCAATCGCAACAAGCTGCCTTGCCCATGGAATGGAAATGAAACTTGGATCTGATA
GCCGAGCACTCCAGTTCGCGGCTTATACGTTCGATATTTGTATCGCTGAGATTTTTACGACTTTGATATTCGGCGGCTGT
GTCTGTGTCCCTTCGGAAGACGATCGACGTAACGCCTTATCCGAAGTAATCAACAACAATAACATCAACTGGGCTCAACT
CACGCCGACTGTTGCTCGTCTACTAGATCCCAGCACGGTCCCTTCTCTGAGAGTGCTCGTCCTTGGAGGAGAACGAGTGG
ACGAGGCCGACTGGAAGAGATGGGGTGACGATATCGTGAAGGTGAACGTTTACGGCCCGACGGAGTGTAGCATTTGGTGC
ACTTCGTATTCCAATACCGACCGAGAATTCCGATCGGGAACGATAGGCACGTCAATGGCTTCTTTTAGTTGGGTTACCGA
CCCGGAAGACCACAACAAGCTAGTGCCATTTGGCACAATTGGCGAACTTCTGATAGAGGGACCAATTCTGGCACGCGGGT
ACCTCAACGACATTTCGAAAACTGAGGCTGTTTTCGTTGATGGCCCTCTCTGGCTGAGGCAAGGCAACCGCAACGATGAA
TCCACTCGACGACAGGGAAGACTATACAAGACTGGTGATCTAGTCTACTATGATGCAGACGGTAATCTGGTTTACGCGGG
TCGTAAGGACAGCCAAACCAAGGTGAGGGGTCAAAGAATTGAGTTGGGCGAGATTGAACATCATCTCAACCAATGCATGT
CAGGCATCAAACAAGTTGCGGCTGAAGTCATCCTACCATCAGGTGACCAAGCAAAAGCGATGGTGGCAGCATTCGTGCAG
CTAAGCGAAGAGCCACGCCATGCACTCGTCCAACAGACTTCTAACGGTGACTTGGAAGTGCGAGTCATCTTCCCAACCTA
TCTGGATGAGTTGCTGGTTCAGTGTCTGCCAAAGGACATGGTGCCTGAGGTGTACTTTGCCGTAGCGGAGCTTCCATTGA
CAACATCCGCTAAAGTGGACCGGCAGAAGCTGCGCAAGATTGGCGCCTCCTTTTCTGCACAACAATTGGCCCAGCTTCGA
ACGTATAGCGATGACCCAAAGCGACAACCAGAGACTGAGAAAGAGCAGATCTTGCATCATCTCTGGGCACAGGTCCTAAG
CATTGACGCCAGTTCAATTGGTATGGATGATAGCTTCTTCGATCTGGGCGGCGATTCAATAGCAGCTATGAAGCTGGTGG
GTGAAGCGCGCAGATCAGGAATATACATCACTGTGGCAGTCGTCTTCCAGAACCCCACGCTAGACAAGTTGACCTCTGCA
GCGATCCCTTCAGTCGATGTGTCTAATACCACCATTCCACCTGTTGGCCATGACGGGCATGTTGCACAATCATTCGCACA
AGGTCGGATGTGGTTCTTAGAAGAACTTCACCCTGGTCTTACCTGGTACCTCATGCCGGTTGTTGTGCGTATGAGAGGCC
CGTTGGAGCTGACAGCGCTCCAATCTGCGCTGAATGCAATTGAGAGCCGCCATGAGACCCTCCGGACGACTTTTGAAACC
ATTGGAGATACAAGCATGCAGCTGGTTCATCCATACCATGCTAAGGAGCTGAGTATCATCGACATAGACATCAAAAGCCT
CGAAGAAGTACTGCATCGGGACCAGATAAGTCCCTTTGACCTGCGTAAGGAGGCCGGTTTGCGAGTGTCAATCTATCGCA
TCGGCAGCGAAGAACACGTTCTGTCTGTCATCATGCACCACATTATTTCTGATGGCTGGTCTACTGATGTATTTACGCGC
GAGTTGGGCGCGTTCTATTCAGCCTCAATACGAGGCCACGACCCTCTTGTTCAAGTCCAACCTCTGCCTATTCAGTATCG
AGACTTCTCTGTGTGGCAGAGGCAACAAGCCCAGATCGACAAACACCGGAGTCAGCTCAATTATTGGTTCAATGTGCTTA
ATACCAGCAGACCCGCTGAGCTGCTTTGCGACAAAGCTCGACCAGCTGCTTTATCTGGGGAAGCTTCCAAGCAGACGATC
CAGATCGACGGCCCGCTATACATTCAGCTGCTGCAGTTCTGCAAAGCGAAAGGAGTCACAAAGTTCATGGTGCTATTTGC
GGCTTTTAGAGCTACACACTTCCGCCTCACTGGCCAGAACGATGCCACCATTGGCACTGTGAACGCCAATCGGGATCGAT
GGGAGCTGAAAGATATGATCGGTTTCTTTGTCAACCTACAGTGCCTTCGGACTACGATTGACGCGGACGAATCGTTTGAA
GAACTGGTTCAGCAAGTCTATGAAGCAACAATTGCTTCACTTGCTAACGCAGACGTCCCGTTTGAGAACATTGTGTCCAA
GCTCAAGAACAGCAGAGACTTGTCTCGTCATCCCCTTGTACAGCTTGTCTTTGCTATGCATTCCCAGCGCAACTTGGGAC
AACTGAAACTTGAGGGCCTGGAAACAGAGAGCCTGGATAATGCACCGAAGTCAAGATTTGATCTTGAATTCCACTTTTTC
CAGCAAGAAGACAGCCTGAAGGGTGAAGTTGTCTATTCCACCGACATTTATTCTCCAGAGGCGATCGACAACATGCTGTC
GATCTTCCAAATCGTTCTCGAGGGGTGCCTCCAAGAGCCGAAAGCTGCGATTGCGTCCTTGTCATTGTTATGCGACGTCG
AATTATCGAAGCTCAATAGCATGGGTCTGATCCAGGTTGAAAAGACGGATTATCCACGAGAGTCGAGTGTAGTCGATCTG
TTTCGCCAACAAACTTCCCTCTGTCCGTCCAGGATTGCTGTGAAGGACGCATCAGTAACCATGACTTATACTCAGCTGGA
CAAGGAATCTGACATTCTTGCTCAGTGGTTAGCCAAACAATCGCTCGCTCCGGAGACACTGGTCAGCGTATTAGCAGGGC

GCAGCTGTCAGACTATCGTCGCATTTTTGGCTATTCTGAAGGCGGGTCTAGCATATTTACCGTTCGATGTCAGGGTTCCG
GCCAAACGGATGAGTACAATTCTTGGCTCACTCTCAGGCCCCAAATTTGTGCTTTTAGGCGAAGACGTACAGCCTCCTTA
CGTGGACATTAGTGACATTAGATTCATACGCATCACAGAAGCGTTAGACGAGCAAACTCACGAAGGGTCCGCCTCTCGGG
ATATTGTCAAGCCCACAGCCAATAGCCTTGCCTATGTTATGTTTACGTCAGGGTCAACCGGTCAACCGAAGGGAGCCATG
ATTGAACACCGTGGTATCGTGCGCCTAGTGAGGGACAATAACTTTGTGCAGCATTTGCCGGCTTCGCCAGTCATGGCGCA
CATGACGAATCTGGCCTTTGATGTCTCTACGTGGGAGATATATGCTTCTCTACTGCAGGGCGGGACGCTAGTATGCATCG
ACAGACTGACAGTTTTGGATCCAGAGGCTGTGCTACGAACTTTTCGTCAAGAGCATGTCAGCACAGCCTTCATGACACCA
TCTCTCTTCAGGACTTACGTTCAGCAATTGCCTGCACTGTTCGCTGGTCTCGATATGCTTTGCGTTGGGGGTGAAGCACT
TCACTCGAACGACATCCTTTCTATGACAACACTTCGCACAGGAAAGATCATCAACGGCTATGGGCCAACCGAAAACACAA
CGTTTAACACAACTTTTGTGCTTTCAAGGGAAGGGCAGTACCCGAACGGTGTGCCCATTGGCCGTGCACTTAGCAACTCT
GGCGCCTATGTCATGGATTTGAAGCAGCAGCTTGTTCCGCTAGGGGTCGTTGGAGAGCTTGTAGTCACGGGTGACGGCCT
GGCACGTGGATACACCGACCTTGAGCGCAACATATACCGCTTCATAACGGTACAAATTGGGGGCGAGGTCGTGAAGGCTT
ACCGTACTGGTGACTCAGTGCGATATCGTCCTGCAGACGGCCAGCTAGAATATTTCGGCCGCATGGACGGACAGGTCAAA
ATTCGGGGACATCGCATCGAGTTGGGTGAGATTGAGCACGTTCTCCGTAGCCACGGGTCTGTAAGAGAGGCTGTAGCTGT
GGTGCAGCAGCAGCAAAACGCAGATGAGGCTGCTCGACTAGCCGCTTTTGTCACAGTCTACGAGGGTGACGAATTGGTTG
AGGAAAAGCCAAGTGGTATCGACGAATCGGAGCATGTCGATGTATGGGAAGACCAGTTTGATTCCAAAGTCTATACGCCA
ATCTCCAAAGTGCTTCCTGAGGCCATCGGGCGAGACTTCATCGGATGGACATCTATGTACGATGGCAGCGCGATCGACAA
AGTGGAGATGAACGAGTGGCTAGACGACACGATCGACACGATGCTCAACGGTCATCCACCTGGCAAGGTCCTCGAGGTAG
GCACAGGTACCGGTATGGTTCTTTTCAACCTGGGAGATGGTTTAGAAAGCTATGTCGGGTTGGATCCATCTTCAAGAGCT
GTGGAATTCGTCAAAGACACAGTCAGATCAGTACCTACGTTGGCAGACAAGGTCAGAGTCTACAAGGCCACAGCGACAGA
GATCGATCGACTAGAGCCTATCGATGCAAGCCTTATCGTCATCAACTCAGTCATTCAGTACTTCCCAAGCCTTGAATACC
TTTTCAAAACCACACAACAACTACTGGGACTAGAGAGCGTCTCTACCATCTTCTTTGGCGATGTGCGGTCTTATGCGCTA
CACCGAGAATTTCTTGCAACTCGAGCCATGTTCATGGCTGGCGACAGTGCAGACAGGGCTGAAGTCAGTCGTATGATAAC
TGACATGGAGCTGGTCGAAAAGGAGCTCCTGGTAGATCCAGCCTTTTTCACTGCTCTGCCTGAGCGCCTGCCAGACCAAG
TAGAGCATGTCGAGATTTTGCCAAAGAAGATGAAGGCAACAAATGAGCTAAGTTGCTATCGTTACGCCGCGGTTATCCAT
GTTAAGCCGCGAGATGGGCGAAAGCAAGAGCAGAGGATTCGACACGTGGGGCACGATGAATGGATCGACTTCAGGGAGCA
CAAGCTAGATCGCCAATCTCTCTTGGCACAGTTGCAAAGCAATCCCAGACCATCCACCATGGCCGTGAGCAACATTCCAT
ATAGTAAAACGATCGTGAGTCGGTGTCTGATCGAATCGATAGACAACGCAGTGGCAGAGTTGTCTGATCCTCAAGACTGG
TACTCATCCGTGTGTCAGCGAGCACAGTGCTCTTCATCCATGTCTGCTACTGACCTATATGAGTTGGCGAAGGAAGCCAA
CTGCCGCGTTGAAGTTAGTTGGAGCAGGCAGCACTCACAGTGCGGTGGTATTGACGCTATATTCCATCGATACCCACCGC
GTGGGGGAGAGAACAGGGTGATGTTTCAATTTCCCACCGACCACGCAGAGCGACCCCTACACACTCTGAGTAGCATGCCG
TTACGGCAACAAACCCTACAGAGAATCCAAGGACAACTCCAGGAGATGCTTGACGCTCAGCTGCCTGCCTATATGGTTCC
CCAGACAGTTACGTTCCTCGAAACCATGCCAACCAACCAGAACGGCAAGATAGACCGGAATGCCCTCACACAGCGAACCG
AGATCCAGGTTGCAAAAGGCCAGGAGTTCCAAAGAGAGCTCACTAGGGCAGAATCAAAGATCCAGCAGTTAATCGCGCGC
GTACTTCGCATCGATTCGGACCGCATTGGTCTAGATGATAGCTTCTTTCAGCTAGGTGGAGACTCAATCGCGGCGATGAA
GCTGGTTGCTTTAGCTCGAGACGAAGACATCCGACTCACCGTGGCAAAAATCTTCCAATATCCCAAACTTATCCAGTTGG
CTGCCGTAGCGCAAGAGCATGTTTACGTTCCCAATGACAACATCGTCCCATTTTCTCTCCTGGACGACGAGGTTGATGCA
ACGCAGACACACCACGAGGTGGCAGTCAAGTGCGCCATCGATAGGGGTATCATCGAGGATATCTACCCATGCTCGCCGCT
TCAGGAAGGCTTGATGTCGCTGACTGTGAAGCGGCCGGGGGATTACATCATGCAGACCGTGCTGGAGTTGCGGGAGGAAG
TAGATGAGACAGCCTTCAAGATTGCATGGGAGAAGACGGTTCAGTCTTTCCAAATCCTGCGAACAAGAATTGTCATACAT
GAGACCCTTGGCCTTTTGCAGGCTGTCATAGCGGAAAAGATAAAGTGGGCAGATGCAGACGACCTCGCTACTTATCTAGC
TCGAGACAAGCTGTCATCTATGCAACTTGGCAAGCCGCTTGCACGCTACGGTCTTGTTCGGGACACTCGCAGAGAAAAGA
AGTGGTTCGTATGGACAATTCACCACGCGATATACGATGGTTGGGCGCTCAATCATATCTCAGTGCTGTGCAAACAGCAT
ACAATGGCAGGGAAACCGGGAAAGCAGGTTGGTTTCAACAGTTTCATCAAGTATCTCCGTCAGATGGACGAAGACGCTCT

CGCAGAATACAGGCGAACCACCCTGAGCGACTGCGACGCGAACGTCTTTCCGCCACTAGTATCTGGGGTGCAACAGCCAG
TTGCGGATGCAACAGCGGAATATTGTTGCCCACCACTTCCCAAGAGAACCTCGAATACTACTATCTCGACACTTGTCCGT
GCTGCATGGGCAATCGTTGCTAGCGGCTACACCAGCTCAGACGACGTTGTGTTTGGGGCGACAGTTACTGGTCGAAACGC
ACCAGTGGCTGGCATCGAGTCCTTGGTGGGTCCTGTTATTGCAACAGTGCCTGTTCGCATACGGTTGCAGAGAGACTCGA
CTATACTAGAATTTCTTGAAACAGTACAGAAACAGGCAACTGAGATGATTCCCTTTGAGCAAACGGGCCTACAACGAATC
GCCAAGCTTGGGCCAGACACCGAGCATGCGTGCAATTTCCAGACGCTGCTGATAGTACAGCCTGCAGAGGACGCCTTCCA
ATCAGACGATATGTTTGGGACGTGGGAATTCGGCTCGGGTCTGCAGGACTTCACGACGTATGGGTTGATGGTGCAGTGCA
AGCTGGCTAAAGAGGGTGTCAAAATCACGGCCAGTTTTGATGCGCGTTTGGTCGAACAGTGGCAAGTTGAGAGAATGCTA
GGCCAGCTAAGCTTCGTCATGCAGCAGCTAGCGCGAGGAGATTCAAGAACAAGAGTAATGGATATTGGAATGCTTACACA
AGATGATGAACAGCAGCTCTGGATGTGGAACCAGAGGCTACCACCAGCTATCGATCGCTGTGTCCACGATCTTTACTCGG
ACCAAGCGAAATCACGGCCTGAAGCGGATGCAATCTGTGCTTGGGATGGTGTAATGACGTATAAAGAGCTTGACGAACGA
TCATCGCGGCTAGCGACCTATTTGGTCGACATCGGTGTCAAACCAGAAACCATAGTGCCGTTATGCTTCGAAAAGTCAAT
GTGGATGGTCGTAGCCATGCTGGCTGTGCTGAAAGCAGGGGGTGCCTTTGCGCCCTTAGACCCGAGCCATCCGGTATCTC
GACATCGAGATATCTTTACGCAAACTAAGGCCAACATGATGCTCACATCATCTCAGTACGCCAATCTCTGGTCTGAATAC
ATCCCCACTGTCGTGGAAATCACCGGACACTTTATTGACCAGTTGACGACAAATCCTTACAGCACTGAGACAGCAGTGCA
ACCAGGCAATACAGCGTATGTCATCTTTACCTCTGGTAGCACTGGAGTACCTAAGGGTGTTCAAATGGAACATAAGGCCG
TTTCCACGAGTTGCTCATGTCAAGGACCAGCGTTAGGTATCACGGAGGATACACGGGTTCTTCAATTCGCTGCCTACACC
TTTGATGCTTGCATCTTAGAGATCATCACAACACTCCTACATGGCGCATGTATATGCATACCTTCTGAGACGCAGCGCCG
CGACCATCTCGTCAACACAATCAATACGATGAAGGTAACCTGGGCGCTGTTAACTCCAGCCGTCGCGAGGATTTTAGATC
CACAGAAGATAGTATCGTTGAAGACATTGGTTCTTGGCGGTGAGAAGGTCAATGGTTCAGACTGCGATACTTGGAGCGGC
CGCGTACGACTGATCAACGCCTACGGGCCCACTGAATGTTGTGTCTCCTGTGTCGCAAGTCCCGACATGAAGGGACTCGA
TCCGGAACCCATTGGGAAGCCGATtGCGTCaATTGGCTGGGTGACTAATCCTAACGATCACAATAGACTAGCACCGCTAG
GCGCTGTTGGAGAGCTGTTGGTTGAGGGACCAAACCTAGCCCGCGGCTACCTGGACGATGCGAAGAAGACAGAGACAGCT
TTTGTCCATGATCCTCTCTGGCTGCTTCGGGGCTGTGAAGGCTACAGTGGGCGGCGAGGTAGACTGTACAAGACAGGCGA
CCTGGTCTACCACACTTCAGATGGTGATCTGGTCTATGTAGGCCGCAAAGATGGTCAGGTTAAGGTGCGAGGCCAACGCA
TTGAGCTTGCCGAGATCGAAATTTGTCTCTACCAACATATCTCAGATATCAAGGAGATAGCAGTTGAACTCATCTCGCCG
ACAGGAGGAAAGCCGATGATTGCAGCATTCCTGAAGGCAAATCCCGAGCTGCTCAATGACAAGCTGTCCGACGGGGACTC
TGGAGTATACGTCGTATACCCAGCCCGAGTAGACAACGAGCTATCTCAGCGACTACCCAGAAATATGGTGCCTGAGGTTT
ACTTTGCACTCACCGAATTCCCGATATCAACCTCTGGGAAGATAAATCGAAGACGACTACGCGAGATCGGAGGCTCTTTT
TCGACCGATCAACTAGCGCGTTTACGCACACAGAAAAACGAGAGCTCGGATCGGAAACCGGAAACAAAGCATGAGATGGC
GCTGCAGAAGCTATGGGCTCAAGTGCTGAATATTGAAGCTACCTCTATCGGACTGAACGATAGCTTCTTCCAACTAGGGG
GCGACTCAATTTCGGCTATGAAGCTTGTGAGCGAGGCGCGTAATGTCGACCTAGTGTTTTCTGTTCAGGACGTTTTCCAG
GTGCAGCGACTGGGTCGGCTAGCCAACCGATTGGTCGACCCTCCCACTTCAAGTCACAGTGCAATCACCAAGATTGATCA
TCAGCGACCCGTCCTTCAATCGTTTGCGCAAGGGCGACTCTGGTTTCTAGAACAACTGCACCCAGGACTAGATTGGTATC
TCATGCACCTTGCAGTACGCATCAAAGGTCCTGTCCAACTCCCTGCACTCCAGGCTGCATTACAGGCAATAGAGCATCGT
CATGAAACACTAAGGACAACATTCTCCACCAACAATGGCGAAAGCCTACAGGAAGTTCATCCTTTCTGTGGAGGAAGAGA
ACTCAATGTCATCGACGTTGGTTCGAACGATGACAAGATTCTGCTCGAAGCACTTGAACGGGATCAGAAAACACCATTCA
ATCTTCGGTACGAGCCTGGCTGGAGGATATCAATATATCGTATCAACGATGTTTCTCATGTTCTATCGATCGTCATGCAT
CACATCGTCTCAGATGGCTGGTCAGTTGATGTTCTGAAGAAAGAATTGAGCGCTCTATATGCTTCCGCCATCCGCAACGA
AGATCCCATCTTCTGCTTGCCGCCTTTACCCATTCAATACAGAGACTTTTCTGTCTGGCAGCGACTACCAGAGCAAGCTC
AAGAACATCGACGGCAGCTGGACTACTGGATCAACCAATTAGACGGAAGTCGGCCAGCAGAATTCCTCTACGATAAACCA
CGACCAACGACTCTGTCTGGTAAAGCGGGAACGCAGAGACTCAACATCAGCCATAAACTCTATAACAGGCTGCAAATATt
TGCTAGGCAACGTGGGATGACTCCATTTGTCGTCCTCCTCGCCGTATTCAGAGCCACACACTATCGTTTGACCAACCAAG
ATGATGCAACTATCGCAGTCCCTAATGCGAACAGAAGTCGCCCAGAGCTCGGAGATCTGATTGGGTTTTTCGTCAATATT

CAGTGCATGCGAATGAAGATCCAGGATGAAACCTTCGAAGAGCTATTGCAGCATGCCTACAAGACAGTAGTAGACTCCCT
TGCCAACCAGGACGTACCTTTTGAAAGTATTGTTTCTGCATTGCAAGGGGACAGGGACTCTTCACGCAACCCTCTCGCGC
AGGTCGCCTTCGCTGTTCACTCACAGCAAGATATAGGGAAGCTCGATTTTGAGGGCGTCGGGACAGAAGCCATAGAAGGC
CTAGCGACCTCACGATTCGATCTCGAGTTCCATTTCTTCCAGGAAAAGAATGGTTTCCAAGGCTATATATATTTCTCGGA
GGAGCTGTTTGTCCCGGAGACTATATATTCGCTAGCATCTGTCTTCACCAGCATCCTCGACAACTGTCTCGACAAGCCAG
AGACACAGATCGCCGTAGTGCCACTCATGACCGTTGAGGCTCACACTCAGCTCGACCAAATGGGGCTCCTCCGCATGCAT
CAAACAGCCTATCCCCGGAATTCCAGCATCGTGGACGTCTTTCGCCAGCAAGCCGCTATGCAACCATCAAGGGTGGCTGT
AAAGGACACATCAACAGACTTGACTTACGCACAGCTAGATTCACAATCAGAAAAGCTAGCAAAGTTCCTAGCCACGAAGT
CATTTGCCCCTGAAACAGCAGTTGGTGTTCTGGCACATCGTTGTTGTCAAGCAATCGTTGCTTTCATTGGCATCCTCAAA
GCCGGCTTGGCCTATCTACCATTCGATCATAAGGCGCCCGAAAAACGGATGGAGTCCATCTTCTCGACCATTGAAGGCAA
CAAATTGGTCTTGATCGGCCCAAACATCTCACTGCCAGGTACTGGACCCAAAGATGTTGAATTCGCCTACATTCCCGATA
TCTTAGACGCAGATGAAGATTTTGAGTTTACAAGGAGCGAACTGGATCCTACTCTCAGACCCACTGCCTCTAGTCTGGCT
TACATTCTCTTTACGTCTGGCTCTACTGGTCAACCCAAAGGAGTTATGGTCGAACATCGCGGCATAGTGCGTTTGGCACA
ACATGATCAGATGGAGCACTTCAAGTCTTCCGGAGCGATGGCTCATATGGCAAATCTTGCTTTCGATGGGTCTTCCTGGG
AGATCTACACATGTCTTCTCAACGGAGGTACCCTCGTTTGTATCGATGCGACAACCGTGCTAGACCAGGATGCTTTATTA
CGCGCGTTTACAGAATCCCAGATACGGATCGCTTTCATCACACCTGCTCTGCTGAATTACATCCTGGCGGAATCCCCAGA
TACAATAGGCAACTTGGATACCCTACTTGTAGCAGGAGACAGAGCTGATGTAGACGACGTCTTCAGGGCACGAGATCTTG
TGAGAAACAAAGTCGTTGCCAATGCATATGGTCCaACTGAGAACTCGGTCATGAGCACACTATATATTCTCTCTGAGGAT
GAGAATTGTGTCAATGGTGTGCCTATTGGACGACCTATCAGTAACTCAGCGGCATACGTGATGGATCCAGAACAGAACCT
TGTGCCACTTGGAGTTTTCGGAGAGCTCGTTGTTACTGGAGATGGTGTTGCCAGAGGATATACCGATCCTAGGCGCAATG
TTGATCGTTTTGTGACTGTCACAATCGGACACCAAACAATGCGAGCCTATCGCACTGGGGACTATGTCCGCCAGCGGCCT
CGAGATGGGGAAATGGAATTCTTTGGTCGCATTGATGGGCAAGTAAAGATCCGGGGCAATCGTGTTGAGCTAGGAGAGAT
TGAGACCGTCCTTCGTGGGCATGGGTTGGTGCGCGACGCAGTTGTTGTAGCAGAACAACGAAAAGACAAGAACCAGCGGC
TTTTTGGCTACATCACACTCAAAGAAGACTTCGAGATGCTTAGCGCGCAGAACAGCGATGACGACCAGATACAGCATGTG
AACGCGTGGGAACACCGCTTCAATACCGAGACATATGCTCAAATCGTCGGTATCCAGTCTGAGACAGTGGGACAAGATTT
CATCGGATGGACGTCAATGTACGATGGCACCGACATCGACAAGACTGAAATGAAGGAGTGGTTGGAAGAGACCATCGGTT
CGATCCACGACAAGGTTGGCGGGCAGTTGGGAAACGTTCTTGAGATCGGATCAGGATCTGGCATGATCTTGTTCAACCTC
GGAGATAGTTTGAAACACTATACTGGATTTGAGCCGTCAAGAAAGGCAGTCGAATTTGTTACGGGAACAGCCAGGTCAAT
CCCCTCGCTTGCCAACAAAGTGGAGATGTATAAGGCTACAGCGGCCGACATCAGCAAAGTGGATCAGCCGCTCCAGGCCG
ACTTGGTTGTTCTGAACTCTGTGGTTCAGTACTTTCCCAGCCAAGGATATCTATTCAACGTGGTTCGAGACTTACTCAAG
GTCGATGGGGTCAAAACGTTATTCTTTGGGGACATTCGTTCTTATGCCCTGCGCCGGGAATTCTATGCAGCCAGAGCGCT
CTTCATGGCTGGCGAAAGAGCTAGTCAAAAGGACCTACGACGTCTGGTCGAGGATATGGAACAGATCGAACAAGAGCTGT
TGGTCGATCCTGGCTTCTTTACTTCACTGACACATCGTCTTCCTGATCTGGTCCAGCATGTGGAGATTCAGCCCAAGAGA
ATGAGAGCGACAAACGAGCTTAGCTCCTACCGCTACACGGCAGTCGTTTACTCTCGATCCCGGGAGCCACCGTGCGGAGG
ACTCCGAACTATCCCTGACAATGAATGGATCGATTTCCAGGAACAGGGCCTCAACAATGACTCTCTCCAGCAGCGCATCA
AGGATGTTTCAAGCACACATCCTCTAGCCGTGAGCAACATATCCCACACCAAGACGCTCTTTGGAAACTGCCTGCTAGGT
GCACTGGGCGACGGAAAGGCCAGAAAGCCAGTCCACACGGATTGGACTGCTCACATAAATCGACAAGCCAAGGGGATACC
AAGCCTCTCTGCCGTTGACCTTGACGAGATGGCTAAAGCAGCAGGCTGTCAAGTGCGGATCAGCTGGAACAGACAATACT
CACAGCATGGCGGCCTGGATGCTATATTTTACCCTCGTCAAATCAATGGTGGGAGTGACAAGGCAGGGGTGATGTTTTCA
TTCCCAACCGATCATGCCGAACGTCGACGACAGACCTTGAGCAACAAGCCAATGCGACAGCAACTGGTCAAGGAAGTCCA
ACAGCAACTGGATGAGCTTGTCAAAGTTCAGCTACCTTCTTACATGGTTCCGCAATCTATCCAAGTGTTGAATCAACTTC
CGATCAACCAAAATGGTAAAGTAGATCGGAAAGCTCTCATACAAAGAACAAGAACGCAGACAGAAGTCAGCCAGGGAGGT
CTGCAACGGGAACTAAGCACTGCAGAGCTCAAAGTACAGCGCATACTATCGCGGGTACTTGGAATCGAAGCTAGTCGTAT
GGGGCTAGAGGATAGCTTCTTCCAACTGGGTGGGGACTCGATTGCTGCTATGAAGATAGTCGCTGCAGCGCGCGAAGAAG

AGATTCACCTTACTATCGCAAACATCTTCCAGCACCCTAAGCTCGTCAACCTGGCGACTGTGGCTCAGTTTTCTCAACAT
GAAGGTGAACAAAAGTCCATTCAGCCGTTCTCCCTTCTCTCGACAACCCAAAGAGACTATCTTCTTCACGCCATCCCCGA
GAATACCTCCAATGTGAATGGAAACGACATCATCGACATCCTTCCAACGACATGGATGCAGAATCTTTTCATCTCCCGGG
GTGTCAACATCCAACCCCTGGCGTTCAATTACTTCTTCCTCAATTTGGGGACCCGCGTTGACGCCTCCCGCCTCCGAAGC
AGCATCCCCACTCTCGTCCAACAATTCTCCATACTCCGAACAAAGTTTGTTTACGTCGACGGGGTACTTTGGCAAACTGT
TCTTCGCAAACCACACGTACCTTTTACGGAGTTTCATCTTGACATGTCTCTTGAAGAAGCAGCTGATACTGTTTGCTTGG
AGGACAGTCGGACCACGGATCCCTTGGAGTTGGCTACTGCCTTCATGCTGATACGAGGCACCTCCAATGAACATCTTCTG
GCAATTCGCATCACGCACGCTCAGTACGACGGTGTTTGTTTCCCGTCATTCGTGAAAGCACTTTTTGCCATCTACTCCGG
AAAGTCTGTGGAGCCTGCGCACAACCACTCGACCTATCTGGCATATACTCGGGAAAGAAAATCAGTCTCAGCCCTGCACT
GGCGGGATGTCCTGCACGGTTCGCGAATGACCAAAGCCACCCCGTTGCTCAGTCCAAGCATTCGACATGGCATGATTCCA
GTTGAGGTTCAGACAGAAAGCATCATCGGCATGCCTCACGTCCCGACTGGTCTTACTCTGGCTTCGCTCGTCAGCGCCGC
CTGGGCAAAGGTTCTCTCACAAATCACTGGAGAAGAAGATGTCGTCTATGGATACATGGTTGCTGGCCGCAATGCTAACA
TCCCGGCCATCACCAAGATTGTCGGCCCGTGTCTAAACATCATCCCAGTTCGCGCACGGTTGCATGCAAAGACAACTTCG
ACGGAACTAATACGTTCAATTCAAGAACAGTACATCGCACTCGGCGAAGCAGATTCGATGGGTTTTGATGAAATAGTCCG
CACCTCTACAGACTGGCCGGCCGATACAGAGTACGACTCCGTGTTCCAGCACCAGAACCTGAATGAACATCCCGAATTCG
ACTTTGAAGGCACCAGCTCTAGACTCCATTGGTTTCAGAATCCCGATTCGGTGCCTTGCATCCTTACTGTGGTGTCCTAC
CCGCTTGAAGACGGGCTTAGGATTGTGGTTAGGGGTAACGAACACATCATAACGCCAGAGAGTGCGGAGAGAATCAACAA
ACTGCTCTGTGAGACGATCGGGGCGCTGTCTTCTTCGCTCCAGTAA

Figure S3. >Seq1 (organism = *Alternaria alternata*) tentoxin synthase (TES) gene of *A. alternata* ZJ33, complete cds.

ATGGAGCCTGAGCCTCTAGACCCAAGGGCAATCGGTTTTATGCGTCGAAACCTTGCAATGCTGCGACAAACCCCAATGTC
GGTAGTGCTTGCTGGTCTCATCCTCTTAGCATCTATATATTTCCCACGCTTCATGTTCAAGTCGCAGATGGCGAGATTTC
CACTCATCCTAGAGCATCTAAGTAGCGAGCAGCGGCGAGCAAGGTTTCTCGTAGGAGCAAAGGCACTCTACAAGGATGGC
TCTCAGAAGGTATTTCGACATAAGCAGATACATTTCATTCTATTAACTCGCTCCTAGTTTAGGGGAATGGCTTACAGAAT
GCAAACTTTAGATGGTAAGCGAGGTCTGACAGTACTACAATTATTCTTACTAAATCCTTCTAGAGCAACAGATTGTCCTG
CCTCTTTCCGCATTGACAGAACTTCGCAAAGCCCCTGAGGAGGTGCTATCCTTCTACGACATGTTTTCCAAAGTATGTAA
TCCATGCGACATGAGTGGCGTAGTTGCTCACGCGCACCCAGGGCCTGGAGGAAACCTACACTTATGTCGACATCAAATTT
CTACCCGAGGTTGTCGATACAATCAAGAGGGATCTTACACCCAAGCTGAGTAAGCATGAACATGATGCTCCAGTATGCGT
ATCATGCTAATGAAAACAGCGAAAATCACCTCAAAGATTTGTGATGAGGTTGATGCTGCACTAGACACATATCTCCCCTC
CCACGGCAAGTGGACGGAGATCAATGTGTCAAAAACAGCTCTGGATATTATTGCAAAAGTCTCAGCCCACCTCTTCATCG
GAGGCGATGTTGCCAACGACCCGGGTTATCTCGAATGTACAAAGAACTTTACCGTCCATCTGGGTGAGGCCACCAGAGCA
ATCAAAGTCACGCGGGTGTGGCTCAGACCCTTCCTAGCTCCCCGACTGCCAGAGGTAAAACGTCTTCTCGAGACGAGGAC
AAAGCTAAGAAGCTATGTCAAGCGTGTGATCGAAGAGAGAGAGGCCAAGATCAAGAACCCGGACTGGGTACCACCGGACG
ACATGATGCAATGGCTCCTTGATCGAGCTGACCGGCAAAAGGACACGTTGGAAGACTGCACAGCTGCCCAAGTTCTTCTT
ATACTCGGAACGATCAATGCATCGATGCAGACTTTGATTGCCATTTTGCATACGCTAGCAGTGACTCCCGAGTATGTCGA
GCCGCTTCGAGAGGAGATTCGAAATACTTTGAACAGCGACGGATCGATTCCAGTCAGTGCGATGAAGGAGTTCGGGAAAA
TGGATAGTTACTTCAAGGAGGTGGGCATGCATTTTCCGGTCATTATCGGTATGTGATTTCCTCGTTCCTTTGCTTGCTCG
ATCTCGGTTCTTACGCAAAGCTAAACAAAACTTGCAACAGAACCGTATTTCCGCCGGGTTCGCAAGGGCTTCACTCTGTC
AAACGGGCAGTACCTGCCCCCTGGCGTAGCTATAGTGATAGCGAACCCGCTCGTCACAGACTCCAAGTACGATACGTTTG
ACGGCTTTCGACACTACAAGTTACGAGAGGCCAGCGCGCAAAAGGATAAGCCTAACCATCGATGGCTTATTGCAAATGAA
ACTGAGTTCCGATGGGGCTATGACAACCATGTCTGTCCTGGCAGATTCTATGCGCATAATCTGCTCAAAATTATCTTTGC
TAGAATAGTCGAAAATTACGATATCAAGATGCCAGGGAATGTTGAGGGAATAGAGGCTAGGTATCCAATGGTCGAGCACG
GGAATGTTGTTATGGAACCAAGAGACAAACCTCTTCTACTCAGAAGGGTTAAGAGCCATCAGGGTCACGTAGATTGA

Figure S4. >Seq2 (organism = *Alternaria alternata*) cytochrome P450 (TES1) gene of *A. alternata* ZJ33, complete cds.

GAAGTAGATGAAGCACATGTGCATAGTAAGCAAAGCTGCACGCCTTCCAACCCTCCATTTTCCCCCACATAGCATCATGT
GCTCTCTCAACAAGATTTCCTCCGTATCGAGCTAGTCTTCGCATGCATAAGCATCTGTCTGTACACGTCAATCTACGTGA
CCCTGATGGCTCTTAACCCTTCTGAGTAGAAGAGGTTTGTCTCTTGGTTCCATAACAACATTCCCGTGCTCGACCATTGG
ATACCTAGCCTCTATTCCCTCAACATTCCCTGGCATCTTGATATCGTAATTTTCGACTATTCTAGCAAAGATAATTTTGA
GCAGATTATGCGCATAGAATCTGCCAGGACAGACATGGTTGTCATAGCCCCATCGGAACTCAGTTTCATTTGCAATAAGC
CATCGATGGTTAGGCTTATCCTTTTGCGCGCTGGCCTCTCGTAACTTGTAGTGTCGAAAGCCGTCAAACGTATCGTACTT
GGAGTCTGTGACGAGCGGGTTCGCTATCACTATAGCTACGCCAGGGGGCAGGTACTGCCCGTTTGACAGAGTGAAGCCCT
TGCGAACCCGGCGGAAATACGGTTCTGTTGCAAGTTTTGTTTAGCTTTGCGTAAGAACCGAGATCGAGCAAGCAAAGGAA
CGAGGAAATCACATACCGATAATGACCGGAAAATGCATGCCCACCTCCTTGAAGTAACTATCCATTTTCCCGAACTCCTT
CATCGCACTGACTGGAATCGATCCGTCGCTGTTCAAAGTATTTCGAATCTCCTCTCGAAGCGGCTCGACATACTCGGGAG
TCACTGCTAGCGTATGCAAAATGGCAATCAAAGTCTGCATCGATGCATTGATCGTTCCGAGTATAAGAAGAACTTGGGCA
GCTGTGCAGTCTTCCAACGTGTCCTTTTGCCGGTCAGCTCGATCAAGGAGCCATTGCATCATGTCGTCCGGTGGTACCCA
GTCCGGGTTCTTGATCTTGGCCTCTCTCTCTTCGATCACACGCTTGACATAGCTTCTTAGCTTTGTCCTCGTCTCGAGAA
GACGTTTTACCTCTGGCAGTCGGGGAGCTAGGAAGGGTCTGAGCCACACCCGCGTGACTTTGATTGCTCTGGTGGCCTCA
CCCAGATGGACGGTAAAGTTCTTTGTACATTCGAGATAACCCGGGTCGTTGGCAACATCGCCTCCGATGAAGAGGTGGGC
TGAGACTTTTGCAATAATATCCAGAGCTGTTTTTGACACATTGATCTCCGTCCACTTGCCGTGGGAGGGGAGATATGTGT
CTAGTGCAGCATCAACCTCATCACAAATCTTTGAGGTGATTTTCGCTGTTTTCATTAGCATGATACGCATACTGGAGCAT
CATGTTCATGCTTACTCAGCTTGGGTGTAAGATCCCTCTTGATTGTATCGACAACCTCGGGTAGAAATTTGATGTCGACA
TAAGTGTAGGTTTCCTCCAGGCCCTGGGTGCGCGTGAGCAACTACGCCACTCATGTCGCATGGATTACATACTTTGGAAA
ACATGTCGTAGAAGGATAGCACCTCCTCAGGGGCTTTGCGAAGTTCTGTCAATGCGGAAAGAGGCAGGACAATCTGTTGC
TCTAGAAGGATTTAGTAAGAATAATTGTAGTACTGTCAGACCTCGCTTACCATCTAAAGTTTGCATTCTGTAAGCCATTC
CCCTAAACTAGGAGCGAGTTAATAGAATGAAATGTATCTGCTTATGTCGAAATACCTTCTGAGAGCCATCCTTGTAGAGT
GCCTTTGCTCCTACGAGAAACCTTGCTCGCCGCTGCTCGCTACTTAGATGCTCTAGGATGAGTGGAAATCTCGCCATCTG
CGACTTGAACATGAAGCGTGGGAAATATATAGATGCTAAGAGGATGAGACCAGCAAGCACTACCGACATTGGGGTTTGTC
GCAGCATTGCAAGGTTTCGACGCATAAAACCGATTGCCCTTGGGTCTAGAGGCTCAGGCTCCAT**CTTTGGAGTGTATCAG**
TCTTCTCCGGAAAGAAGAGGAAAACTGTCGTATTGGAACTACCAGAAGTCACAGAATCTCTTGGGGCAAAAGTAATTTAT
GTTGAAAGATACTTTCAGCATTATGCGGAGTTGACAAGTCTACATGCAGTGCAGACTAGCACACCCAATCTCGTAAAGAT
CGAGATGGAGAAGCCCCCGTTATCTATTGGTTCGGATTCGTTTATCTGCGGTTCATCGTAACATCCAAGATATACTAGGC
TGGAATGAGGAGTTACGGTCAAAAGAAGAACACATACGTGTCGCACTGGCTGGGCTATGCTTGATGACATGACGCGTAAC
TTCGCTACACGGTACATTTAGTAGATGTGCTCGGAAACTTTATTTGGTGCCGAAACCTGCATCGGAAAGGTGCAGCCGTT
GCGTGTGAGTTTGTAAGTGGTTGGGTTGACCGTAGTTACGCCGGCTGAGGTACCGAGTCTGATCGATGATGGGCATCTTT
GATTTCCAATTCTCGTCTGCATCGCGACTATTTTCGGGTAATACGGGGTAAGACATCCCGCACGGCCCGAAAAGGGACGA
GTCGAGCCGCTGGGGTAATCAGTGCGGGAGGACCCCATTCGTGCGTCAGTGGTGGGGGAAATGGTTTCCTATCCTTTAGC
GTGCTTACAAGCGGTGGGTTTTCGGACAATGTGCATTCGAGAATCCACGTTATACTCTTGGACTATATTTTTAATCCCTC
TTTCGAAGCCGCAAGTGCCGGATGATGGACATAGTATAGACCCGCAAAGAGATCTCTCTCAGAGGTCTAAGGAAGCCGTC
CCTTCTAGAAAAGCTTCTTTCGGGCAACCATCATGAAATCTATATGGCGAGGATATCGATGAACAAGTCGAAACTTTGAC
TGCCGAGGAGTCGCTACGTTTTGCCAAACTTAGAAAGGGAGCTATAAGCATGTCGACATATCGTTCCCTAATCACTCAAA
CCTCCGCGTGTCCACAATGCAAGCGTATCATGTTGTAAGAAAAAGTACGTGCGATGTCCGACTTGATCTATAGGGCCATA
GGCTACTTTCTCGGTGGAACGCGTAACACTCGCTGACACGAGATGGTCCGAATAGTAAGGGTGCTTCATGTATCCCAAGC
ATCTAGATTAAGAGGAGAAATCTTAGCGGCAGACTTAGGCCATTGTAGCCAAGTGGACGATGGAGGAGGCAGTGAGTTTC
GTCAGGTAGATACCTTGGTGTTGTATCTAGATTGTACAAATCTACTAGTCTCGATCTGTGAAGCGACGCCTGTGAGCTTA
TCTTCTGACATAAATATGCTCGAAAAATAGTCCTGAATCTCAAATTACAGCTTTTCATACACTGAGATCAGCTGACTCAT
CATCTTCCCATCTGAATTCTGTTCCACGGAAACTTCATTCAACAAGAGCCGACATGCCGGGATCGCTACTGTTTGACGTC
AACAAACTCGATGTCGAGAAGATATGGGAGAGAAACCGCG

Figure S5. DNA sequences between *TES* and *TES1* (**red**).

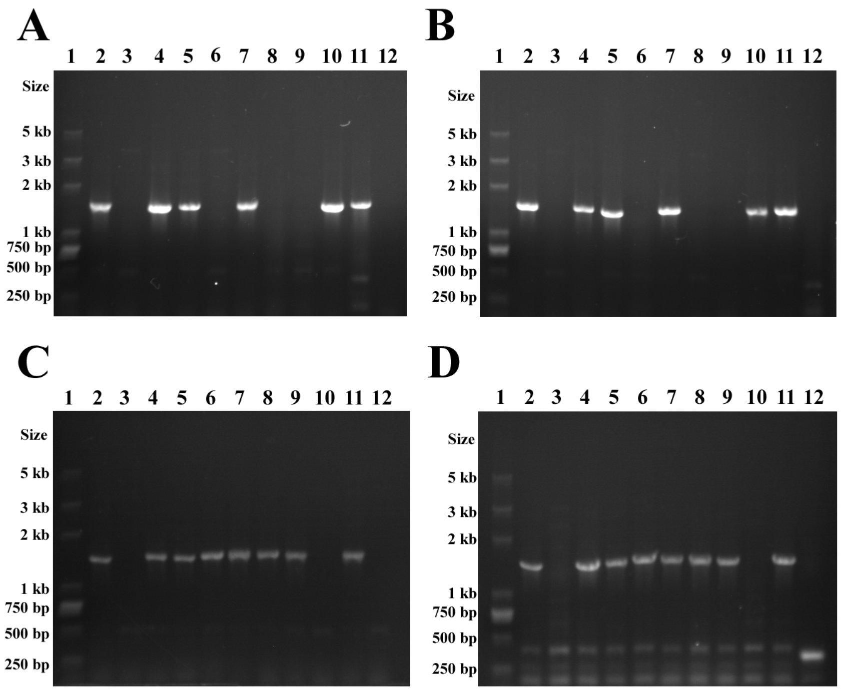

Figure S6. Verification of *TES* or *TES1* disruption by PCR analysis. lanes 1: DNA ladder markers; lanes 2–11: transformants; lane 12: wild-type strain ZJ33; (**A)** verification of *TES* disruption by PCR amplification using primer pairs F1 (TES-5for) + R1 (Hyg-5rev); lane 2, lanes 4–5, lane 7 and lanes 10–11: *TES* null mutants; lane 3, lane 6 and lanes 8–9: ectopic transformants; (**B**) verification of *TES* disruption by PCR amplification using primer pairs F2 (Hyg-3for) + R2 (TES-3rev); lane 2, lanes 4–5, lane 7 and lanes 10–11: *TES* null mutants; lane 3, lane 6 and lanes 8–9: ectopic transformants; (**C**) verification of *TES1* disruption by PCR amplification using primer pairs F3 (TES1-5for) + R1 (Hyg-5rev); lane 2, lanes 4–9 and lane 11: *TES1* null mutants; lanes 3 and 10: ectopic transformants; (**D**) verification of *TES1* disruption by PCR amplification using primer pairs F2 (Hyg-3for) + R3 (TES1-3rev); lane 2, lanes 4–9 and lane 11: *TES1* null mutants; lanes 3 and 10: ectopic transformants.

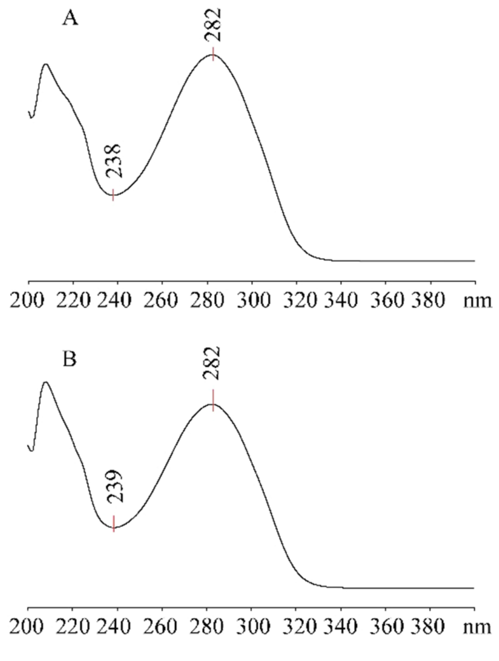

Figure S7. Absorbance spectrum of standard tentoxin and tentoxin from *A. alternata* ZJ33. (**A**) Standard tentoxin (Peak 1); (**B**) Tentoxin (Peak 1) from *A. alternata* ZJ33.
